# ^177^Lu-DOTATATE peptide receptor radionuclide therapy (PRRT) in metastatic phaeochromocytomas and paragangliomas (mPPGL): a single centre retrospective analysis of experience at an ENETS Centre of Excellence

**DOI:** 10.1530/EO-25-0019

**Published:** 2025-10-21

**Authors:** Kalyan Mansukhbhai Shekhda, Eleni Armeni, Yiwang Xu, Manfredi D’afflitto, Aimee Hayes, Dalvinder Mandair, Dominic Yu, Ann-Marie Quigley, Shaunak Navalkissoor, Gopinath Gnanasegaran, Ashley B Grossman, Martyn Caplin, Christos Toumpanakis, Bernard Khoo

**Affiliations:** ^1^ Neuroendocrine Tumour Unit, ENETS Centre of Excellence, Royal Free Hospital, London, UK; ^2^ Department of Diabetes and Endocrinology, Royal Free Hospital, London, UK; ^3^ Department of Radiology, Royal Free Hospital, London, UK; ^4^ Department of Nuclear Medicine, Royal Free Hospital, London, UK

**Keywords:** phaeochromocytomas, paraganglioma, ^177^Lu-DOTATATE, peptide receptor radionuclide treatment, health related quality of life

## Abstract

**Introduction:**

^177^Lu-DOTATATE peptide receptor radionuclide therapy (PRRT) represents a possible therapeutic option for patients with metastatic inoperable phaeochromocytomas (PCC) and paragangliomas (PGL) who demonstrate adequate somatostatin analogue binding on molecular imaging. We describe treatment outcomes in our cohort of patients stratified according to germline pathogenic variants (PV) in succinate dehydrogenase (*SDHx*) subunit-encoding genes.

**Methods:**

In this retrospective analysis, we evaluated 20 patients with metastatic PCC/PGL who underwent two or more cycles of ^177^Lu-DOTATATE therapy. Clinical, radiological, and biochemical responses were assessed 8–12 weeks after the final PRRT cycle. We describe overall treatment efficacy at follow-up after stratifying according to the presence of germline *SDHx* PV. Radiological progression was quantified based on the sum of the longest diameter (SLD) of the target lesion. Progression-free survival (PFS) and overall survival were estimated using Kaplan–Meier survival analysis. We also aimed to investigate the impact of PRRT on health-related quality of life (HRQoL), as assessed using the EORTC QLQ-GINET21 questionnaire.

**Results:**

After a median follow-up of 29 months, we confirmed stable disease in 12 patients (60%), a partial response in one (5%), and progressive disease in seven patients (35%). The absolute mean difference in SLD was +5 ± 12 mm for bone lesions, −4 ± 6 mm for peritoneal, +8 ± 14 mm for liver lesions and −1 ± 5 mm for lymph nodes (paired *t*-test *P*-value 0.273, 0.741, 0.208 and 0.826, respectively). Thirteen patients (65%) had received two or more previous lines of treatment. The overall median PFS for the entire cohort, PGL patients, *SDHx* positive and negative groups was 24 months (95% CI: 9.9–38.1), 18 months (95% CI: 8.4–27.6), 24 months (95% CI: 11.9–36.0) and 18 months (95% CI: 0–48), respectively. No grade 3/4 cytopenia or nephrotoxicity was observed. Overall, HRQoL improved after PRRT, as evidenced by the progressive decline in overall symptom scores in the QLQ-GINET21.

**Conclusion:**

^177^Lu-PRRT appears to be an effective therapy with a good safety profile for patients with metastatic PPGL. It also appears to improve HRQoL in patients with metastatic PPGL. Further studies are needed to explore the most effective treatment modalities in this group of patients and their sequencing.

## Introduction

Phaeochromocytomas (PCC) and paragangliomas (PGL), collectively referred to as PPGL, are rare tumours that originate from neural crest cells and have the potential to become metastatic (1). The prevalence of metastases ranges from 2.4 to 50% for PGL and 2–13% for PCC (2). PCCs arise from the adrenal medulla, while PGLs typically originate from extra-medullary sites such as the head and neck, chest, abdomen and pelvis (1). PPGL represents a significant category of hereditary neoplasms, with up to 40% of cases stemming from identifiable germline pathogenic variants (PVs) (1). Approximately 70% of all patients with inherited PPGL can be categorised into three clusters based on their molecular characteristics and germline PV in susceptibility genes. Cluster 1A tumours commonly exhibit PVs in the Krebs cycle and *SDHx* genes, while Cluster 1B tumours involve PVs in the hypoxia-signalling and von Hippel-Lindau pathways (*VHL*) (3). They are characterised by increased aggressiveness and a relatively higher risk of metastasis (3). In contrast, cluster 2 tumours are associated with PVs in tyrosine kinase-linked signalling pathways (e.g. *RET*, *NF1*) and tend to display less aggressive behaviour (3). Tumours in the rare cluster 3 are primarily associated with *Wnt* signalling pathway involvement and exhibit aggressive and metastatic behaviour (3).

Although cytoreductive surgical resection of the primary tumour is the treatment of choice for metastatic PPGL (mPPGL), this can be challenging due to various factors (3, 4). Inoperable mPPGL are usually offered alternative therapeutic modalities, such as chemotherapy, external beam radiotherapy, tyrosine kinase inhibitors, radiofrequency ablation, and/or radionuclide therapy (3, 4, 5). The response rate and progression-free survival (PFS) of systemic therapy for mPPGL varies depending on factors such as the genetic PV, somatostatin receptor (SSTR) expression, disease extent, and the type of treatment employed. Chemotherapy with cyclophosphamide, vincristine and dacarbazine (CVD) shows a partial response rate of 37% and a PFS of 20 months for PCC and 40 months for PGL, while the response rate of systemic therapy with sunitinib, a tyrosine kinase inhibitor, demonstrated a disease control rate (DCR) of 57% and a PFS of 4.1 months (6).

In recent years, targeted radiotherapies have emerged as a viable treatment option for patients with mPPGL (7). Radiolabelled meta-iodo-benzyl-guanidine (^131^I-MIBG) therapy targets the norepinephrine/noradrenaline (NE) transporter, where the theranostic pair (^123^I-MIBG for imaging and ^131^I-MIBG for therapy) binds and is internalised via the NE transporter and is transported intracellularly to secretory granules (7). While ^131^I-MIBG therapy has been reported to have a DCR up to 85%, it carries significant risk of haematological toxicity (up to 87%) and myelodysplastic syndrome (4%) (8, 9, 10). Peptide receptor radionuclide therapy (PRRT) targets tumours expressing somatostatin receptors (SSTR) not only in gastroenteropancreatic neuroendocrine neoplasms (GEP-NENs), but also in bronchial NENs, NENs of unknown primary, and PPGL (11). It has been approved for regulatory use for grade 1 or 2 GEP-NENs after the successful outcomes of the NETTER-1 trial (12). Evidence has also been emerging for the effectiveness of PRRT in mPPGL, notably in patients with cluster 1A-related PPGL tumours, such as PV in *SDHx*, which demonstrate a high expression of SSTR, suggesting that targeted molecular therapies tailored to these receptors could be particularly effective in treating these tumours (5, 13). However, there are few published data regarding the effectiveness of PRRT in mPPGL (14).

In the present study, we have retrospectively analysed our data from patients with inoperable or progressive mPPGL who received PRRT therapy at an ENETS Centre of Excellence at the Royal Free Hospital in London, UK. Our objectives were to document the effectiveness and safety of PRRT in mPPGL, to gather further evidence of efficacy, to explore the effects on health-related quality of life (HRQoL), and to understand the interaction of germline PV in the genes encoding the subunits for succinate dehydrogenase (*SDHx*) with treatment response.

## Materials and methods

### Population

In this study, we retrospectively examined patients with mPPGL who underwent treatment with Lutetium-177-tetra-azacyclododecane tetra-acetic acid octreotate (^177^Lu-DOTATATE) between 2013 and 2023 at the ENETS Centre of Excellence based at the Royal Free London NHS Foundation Trust, London, UK. For this study, we selected all patients with a previous diagnosis of mPPGL. The original diagnosis was determined based on various factors, including symptomatology, biochemistry, histology, and imaging modalities, including ^123^I-MIBG scintigraphy, ^111^In-diethylenetriamine-pentaacetic acid (DTPA)-octreotide, ^68^Ga-DOTATATE positron emission tomography (PET)/computed tomography (CT), and ^18^FDG-PET (fluoro-deoxyglucose positron emission tomography), contrast-enhanced CT (CECT) and magnetic resonance imaging (MRI). Patients were classified as metastatic if metastases were present at diagnosis (synchronous) or during follow-up after initial surgery (metachronous).

Treatment with PRRT was offered following multidisciplinary review of each case. PRRT was offered to patients with either inoperable metastatic but radiologically avid lesions on ^68^Ga-DOTATATE scans at the time of the original diagnosis, or to those with evidence of disease progression during follow-up after first-line treatment. All patients offered treatment had documented tumour avidity on ^68^Ga-DOTATATE PET/CT at known sites of disease at least equal to or greater than background liver uptake (Krenning score ≥2). Although ^18^FDG-PET scans were not routinely done before PRRT, when there were concerns or the possibility of discordant disease, ^18^FDG-PET scans were performed and, if patients were found to have discordant ^68^Ga-DOTATATE and ^18^FDG disease, PRRT was withheld. When required, patients with symptomatically controlled and surgically treatable primary or metastatic disease, as well as patients with significant bone marrow disease (white blood cell count less than or equal to 2 × 10^9^/L; and a platelet count of less than 70 × 10^9^/L), renal failure (glomerular filtration rate (eGFR) less than 30 mL/min/1.73 m^2^), advanced heart failure, or a World Health Organization (WHO) performance status of 3 or 4 were not considered eligible for treatment.

For the purpose of this study, we documented information on patient demographics including age, sex, tumour type, the presence and extent of metastases, functionality of the tumour, operability, genetic background, and Radiological Evaluation Criteria in Solid Tumour (RECIST) 1.1 assessment pre-PRRT therapy (15).

As this project was a retrospective audit of practice and registered with the hospital audit department (audit registration reference number: RFH_23/24678), ethical approval was not required under the UK Policy Framework for Health and Social Care Practice.

### Genetic testing

Genetic testing and sequencing were performed at the Exeter Genomics Laboratory (Exeter, UK). The coding regions and exon/intron boundaries of the dihydrolipoamide S‐succinyltransferase (*DLST*), fumarate hydratase (*FH*), MYC-associated factor X (*MAX*), malate dehydrogenase-2 (*MDH2*), multiple endocrine neoplasia-1 (*MEN1*), succinate dehydrogenase-A (*SDHA*), succinate dehydrogenase-AF2 (*SDHAF2*), succinate dehydrogenase-B (*SDHB*), succinate dehydrogenase-C (*SDHC*), succinate dehydrogenase-D (*SDHD*), solute carrier gene 25A11 (*SLC25A11*), transmembrane protein 127 (*TMEM127*) and Von-Hippel-Lindau (*VHL*) genes, and exons 5, 7, 8, 10, 11, 13, 14, 15 and 16 of the rearranged during transfection (*RET*) gene were analysed by targeted next-generation sequencing (Twist Core Human Exome/Illumina NextSeq). Identified variants were classified according to standards and guidelines of the American College of Medical Genetics and Genomics and the Association for Molecular Pathology (ACMG-AMP) (16).

### PRRT therapy administration protocol

As per our local protocol, before administering PRRT therapy, imaging scans were reviewed to confirm uptake on ^68^Ga-DOTATATE PET/CT at known sites of disease at least equal to or greater than background liver (Krenning score ≥2). The treatment was carried out in a protected isolation room located in the oncology unit at Royal Free Hospital. Before the administration of ^177^Lu-DOTATATE, oral anti-emetic medication (ondansetron 4 mg) was administered. Renal protection was implemented using standard amino acid infusion (2.5% lysine and 2.5% arginine in 1 L of 0.9% NaCl: infusion of 250 mL/h) infused over a period of 4 h, started 30 min before the administration of the radiopharmaceutical via a secondary pump system. Gelofusine 500 mL over 3 h before PRRT therapy was given to patients whose eGFR was between 30 and 60 mmol/L. The administered tracer activity of ^177^Lu-DOTATATE was 6.9–7.9 GBq, except for one patient with mild thrombocytopaenia (baseline platelet count of 87 × 10^9^/L) who received a reduced tracer activity of ^177^Lu-DOTATATE (3.7 GBq). Blood pressure was monitored regularly during administration of PRRT therapy. All patients underwent a ^177^Lu-DOTATATE post-therapy uptake scan at 4 h after administration of PRRT therapy. According to the standard treatment protocol, this involved administering 3–4 cycles of therapy with 10–12 week intervals between each treatment. In the event of significant toxicity, fewer cycles were given, as noted below.

### Follow-up and assessment of treatment efficacy

Patients’ symptoms and adverse effects were evaluated after each cycle and 3 months after the final cycle of PRRT therapy. Routine blood tests, including haematology, liver, renal, thyroid function tests, as well as tumour markers (e.g. plasma free metanephrines (PFMN) and plasma free normetanephrines (PFNMN)), were compared before and after therapy. Elevated PFMN and PFNMN were defined as >3 times the contemporaneous upper reference limit. The follow-up was terminated only if the patient died or received palliative care, in which case only information regarding the patient’s final status was recorded. Restaging cross-sectional imaging (CECT or MRI) was performed between 6 and 12 weeks after the last dose of PRRT therapy, and subsequently every 4–6 months until November 2024 or death, to assess disease activity and determine if the disease remained stable or had progressed. The scans were reviewed by experienced radiologists. Routine restaging ^68^Ga-DOTATATE PET/CT scans were not performed unless there were concerns of disease progression on cross-sectional imaging. In those circumstances, cases were discussed in MDT meetings and appropriate investigation and treatment plans were made.

The efficacy of the treatment was assessed based on clinical, radiological, and biochemical evaluations. The clinical assessment involved assessing catecholaminergic features and changes in anti-hypertensive medications. Radiological evaluation included CECT and MRI and these were evaluated using RECIST 1.1 criteria (15). Biochemical evaluation was based on PFMN and PFNMN levels, which were compared before the commencement of PRRT and after the final dose of PRRT.

Anatomical imaging involved the use of one of the following imaging modalities: CECT, MRI, or the CT component of ^68^Ga-DOTATATE-PET/CT scans only if it was found to be of adequate diagnostic quality (17). The CECT/MRI responses were evaluated using RECIST version 1.1. The visual and/or semi-quantitative analysis based on molecular imaging or other quantitative measurement parameters, such as maximum standardised uptake value (SUVmax) and standardised uptake value normalised to lean body mass (SUL), were not used or calculated to assess treatment response. Complete response (CR) was defined as the disappearance of all target lesions, partial response (PR) as at least a 30% decrease in the sum of the longest diameters (SLD) of target lesions (relative to baseline sum) with no new lesions or progression of non-target lesions, and progressive disease (PD) as more than a 20% increase in SLD with an absolute increase of more than 5 mm, or the appearance of new lesions or non-target progression as at least a 20% increase (≥5 mm absolute increase) in the SLD of target lesions (relative to the smallest sum) or the appearance of new lesions (15, 18). DCR was calculated by combining stable disease rate, partial response rate and complete response rate.

Overall survival (OS) was calculated for all patients and was defined as the interval between the first cycle of PRRT and death. Progression-free survival (PFS) was defined as the interval between the date of first cycle of PRRT and the date of the first radiological progression according to RECIST criteria or disease-related death.

### Health-related quality of life (HRQoL)

To assess the impact of PRRT on HRQoL, the EORTC NET-specific questionnaire QLQ-GINET21 was utilised (Supplementary Fig. 1 (see section on Supplementary materials given at the end of the article)). The questionnaire was completed by patients at baseline and following each cycle of PRRT on the day of their subsequent cycle. The QLQ-GINET21 comprises a total of 21 items, 17 of which are multi-item scales distributed across five domains: endocrine symptoms (ED), gastrointestinal symptoms (GI), treatment-related symptoms (TR), disease-related worries (DRW) and social functioning (SF21). The remaining four single items relate to body image (BI), information (INF), sexual functioning (SF) and muscle and/or bone pain (MBP). Response options on the Likert scale ranged from 1 (‘not at all’) to 4 (‘very much’), with four of the items also including a ‘not applicable’ option.

### Treatment toxicity

Minor side-effects according to Common Terminology Criteria for Adverse Events (CTCAE) v5 during and after therapy were recorded with telephone calls after 2–4 weeks of each cycle of PRRT. Significant side-effects of treatment in terms of renal and haematological toxicity were collated and graded according to CTCAE v5 (19).

### Statistical analysis

Statistical analysis was performed using IBM SPSS Statistics (version 23). Categorical variables were expressed in absolute numbers and percentages (%). Continuous variables were expressed as mean value ± standard deviation (SD).

We compared the difference in baseline and follow-up clinical, biochemical and radiological characteristics following PRRT treatment for the total group of patients as well as after stratification for *SDHx*-PV. For this purpose, we used a non-parametric test since some data were missing, especially biochemical analysis, and they were not normally distributed. Differences between the *SDHx* positive and negative groups were assessed using chi-square test for qualitative data and the unpaired *t*-test and/or the non-parametric Mann–Whitney U test for quantitative data. We also estimated post-treatment differences in the sum of the longest diameter (SLD) of the target metastatic lesions, focussing on the most commonly observed metastatic locations.

For the purpose of survival analysis, PFS and OS were estimated for the entire study cohort and according to the carrier status for PV in *SDHx* using the Kaplan–Meier method. The log-rank test was used to compare the PFS in the *SDHx* PV-stratified groups. A *P*-value <0.05 was considered statistically significant.

For the HRQoL analysis, a descriptive approach was employed due to the limited size and distribution of the dataset. Raw questionnaire scores for each visit were standardised using a linear transformation to a scale ranging from 0 to 100 following the EORTC linear transformation equation. On this scale, higher values correspond to more severe or worsened symptoms (20). Incomplete responses were excluded from the analysis.

## Results

**Figure 1:**
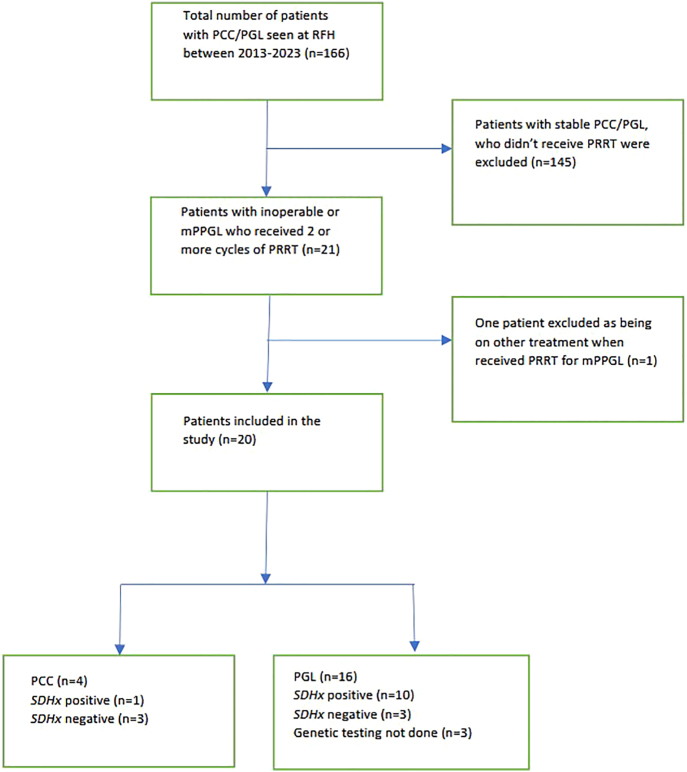
Flow chart of inclusion process of study cohort.

Between 2013 and 2023, a comprehensive cohort of 166 patients with PCC/PGL underwent follow-up at Royal Free Hospital in London. Of these patients, 21 individuals with inoperable or progressive metastatic PCC/PGL received treatment with ^177^Lu-PRRT. However, one patient was excluded from the study due to the concurrent administration of other treatments, resulting in a final inclusion of 20 patients in the analysis. Figure 1 depicts the flowchart of patient selection for the purpose of this study. Of the 20 patients included in the study, three patients (PCC: 1, PGL: 2) were offered PRRT immediately after index presentation with a diagnosis of inoperable metastatic disease based on radiologically avid lesions on ^68^Ga-DOTATATE scans and discussion at the NET MDT.

### Demographics

Of the 20 patients, four had a diagnosis of PCC and 16 had PGL. The sample consisted of 11 male patients (55%) and 9 female patients (45%) with a mean ± SD age of 58 ± 14 years at the start of PRRT. Of the total cohort, 11 patients (55%) had a germline *SDHx* PV (PCC: 1 (*SDH-B*), PGL: 10 (*SDH-B*: 8, *SDH-B* and *D*: 1, *SDH-C*: 1)), while a PV was not found in six patients (PCC: 3, PGL: 3; 30%). Three patients (PGL: 3) did not have genetic analysis. At baseline, eight patients (40%) had elevated PFNMN, while five patients (25%) had normal PFNMN levels. Baseline PFNMN levels were not available for seven patients. The indication for PRRT was radiological progression of disease in 17 patients (85%) or inoperable disease with metastases at the time of diagnosis in three patients (15%). The median number of PRRT cycles administered was 4 with a mean cumulative dose of 24.55 ± 7.77 GBq. The median duration of follow-up from the start of PRRT was 29 months (range 5–134 months). Of the 20 patients, 17 patients had previous treatment for mPPGL. These treatments included resection of primary tumour (*n*: 17, 85%), SSTAs (*n*: 6, 30%), MIBG therapy (*n*: 3, 15%), chemotherapy (*n*: 4, 20%), molecular targeted therapy (*n*: 2, 10%), radiotherapy (*n*: 4, 20%) and ^90^Y octreotate therapy (*n*: 1, 5%). Around half of the patients (*n*: 9, 45%) had received two previous lines of other treatment, followed by five patients (25%) who had received one line of previous treatment. A summary of the patient demographics and tumour characteristics is shown in Table 1. The full analysis of patients’ demographics is provided as Supplemental Table 1.

**Table 1:** Demographic summary of the study cohort (*n* = 19).

Gender, *n* (%)	
Male	11 (55%)
Female	9 (45%)
Age (years)	58 ± 14
Primary tumour, *n* (%)	
PCC	4 (20%)
PGL	16 (80%)
Secretory, *n* (%)	
Yes	10 (50%)
No	5 (25%)
Unknown	5 (25%)
Carrier of *SDHx* germline pathogenic variations, *n* (%)	
Yes	11 (55%)
No	6 (32%)
Unknown	3 (16%)
Previous treatment history for mPPGL	
Surgery of primary tumour	17 (85%)
MIBG therapy	3 (15%)
SST analogues	6 (30%)
Chemotherapy (CVD or CapTem)	4 (20%)
Molecular targeted therapy (sunitinib or sorafenib)	2 (10%)
Radiotherapy	4 (20%)
^ 98^Y octreotate therapy	1 (10%)
Previous number of treatments received	
0	2 (10%)
1	5 (25%)
2	9 (45%)
3	2 (10%)
4	2 (10%)
^177^Lu PRRT characteristics	
Median number of cycles	4 (2–4)
Cumulative dose of treatment (GBq)	24.55 ± 7.77
Median follow up (months)	29 (5–134; IQR = 58)

*n*, number of patients; M, male; F, female; SD, standard deviation; PCC, phaeochromocytomas; PGL, paragangliomas; PRRT, peptide receptor radionuclide treatment; GBq, gigabecquerel; *SDHx*, succinate dehydrogenase subunits A–D; IQR, interquartile range.

### Radiological responses to treatment

Of the total cohort, there were no complete responses noted following PRRT; one patient showed a partial response (5%), SD was confirmed in 12 patients (60%) while PD was recorded in seven patients (35%). Of the cohort of patients with PD at baseline (*n*: 17), one patient showed a partial response (6%), SD was confirmed in nine (53%) and seven patients (41%) exhibited PD. The most common site of metastases at the start of PRRT was bone in 12 (63%), followed by lymph nodes in 6 (32%) and the peritoneum in 6 (32%). The mean SLD at baseline was 101 ± 59 mm, while the mean SLD at follow-up was 108 ± 66 mm (non-significant). Of the 20 patients, seven (37%) developed new lesions on follow-up imaging. Radiological evaluation using RECIST assessment in the study cohort is described in Table 2. Detailed assessment including the site of metastases and RECIST assessment of the total cohort is provided in Supplemental Table 2.

**Table 2:** Radiological features and RECIST assessment of the study cohort.

Indication for PRRT, *n* (%)	
Progressive disease	17 (85%)
Inoperable disease	3 (15%)
Total number of patients	20 (100%)
Site of metastasis, *n*	
Lungs	3
Liver	5
Adrenals	3
Lymph nodes	7
Bones	11
Peritoneum	6
SLD (mm) (mean ± SD)	
Baseline	101 ± 59
Follow up	108 ± 66 (*P* = 0.73)
SLD change percentage post PRRT	6 ± 20
Non target status post PRRT, *n* (%)	
Progressed	4 (20%)
Stable	15 (75%)
Disappeared	1 (15%)
New lesion	7 (35%)
Post PRRT RECIST assessment, *n* (%)	
Stable disease	12 (60%)
Progressive disease	7 (35%)
Partial response	1 (5%)

*n*, number of patients; RECIST, radiological evaluation criteria in solid tumour; PRRT, peptide receptor radionuclide therapy; SLD, single lesion diameter; SD, standard deviation; mm, millimetre.

**Figure 2:**
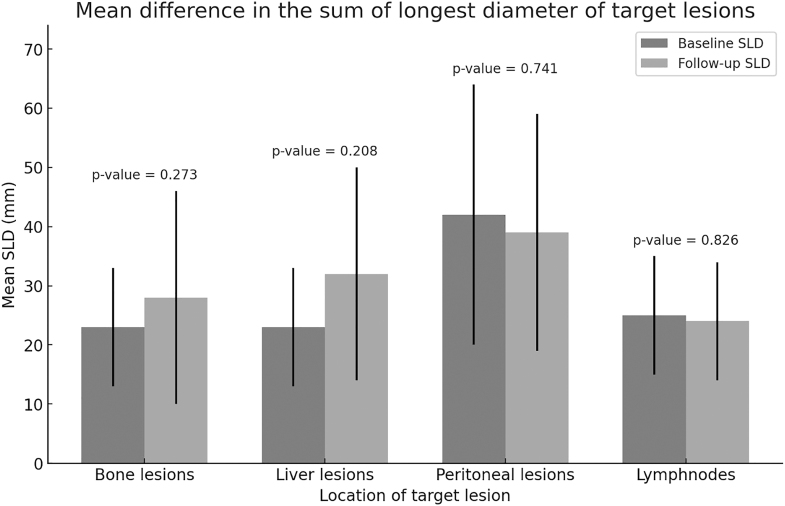
The mean difference in the sum of the longest diameter of the target lesions at baseline and follow-up.

We also compared the changes in terms of the sum of the longest diameter of target lesions, focussing on metastatic disease. Focussing on the sites of the most prevalent metastatic disease, we observed the following changes, which are also shown in Figure 2:

- For bone lesions, the mean SLD was 23 ± 10 mm at baseline versus 28 ± 18 mm at follow-up (absolute mean difference, +5 ± 12 mm, *P*-value for paired sample, 0.273). With regards to the treatment effect on bone lesions, a documented increase in size of bone lesions (>5 mm compared to baseline) was evident in five patients, a reduction in size (more than 30% reduction from baseline) was evident in one patient and stability was observed in five cases.
- For peritoneal lesions, the mean SLD was 42 ± 22 mm at baseline and 39 ± 20 mm at follow-up (absolute mean difference, −4 ± 6 mm, *P*-value for paired sample 0.741). With regards to the treatment effect on peritoneal lesions, three patients had a reduction in the size of the peritoneal lesions (>5 mm reduction compared to baseline) while two patients had stable peritoneal lesions post-PRRT.
- For liver lesions, the mean SLD was 23 ± 10 mm at baseline versus 32 ± 18 mm at follow-up (absolute mean difference, +8 ± 14 mm, *P*-value for paired samples, 0.208). With regards to the treatment effect on liver lesions, three patients showed disease progression (>30% increase in SLD compared to baseline) and two had stable liver lesions.
- For metastases to lymph nodes, the mean SLD was 25 ± 10 mm at baseline versus 24 ± 10 mm at follow-up (absolute mean difference −1 mm ± 5 mm, *P*-value for paired sample 0.826). With regards to the treatment effect on lymph node size, four patients had stable size post-PRRT, whereas one patient had >30% reduction in size of lymph node and two patients had >5 mm increase in the size of the lymph nodes.

### Anti-hypertensive treatment

Of the entire cohort, 12 (60%) patients were on treatment with anti-hypertensives e.g. β-adrenoceptor blockers, α-adrenoceptor blockers, angiotensin-converting enzyme inhibitors and/or calcium channel blockers before their treatment with ^177^Lu-PRRT. Of the eight patients with elevated PFNMN at baseline, anti-hypertensive doses remained unchanged after the completion of treatment in five patients (62.5%), an increased number of medications was required in two patients (25%) while a temporary increase in the dose of medications was needed in the case of one patient (12.5%).

### Toxicity

No new CTCAE grade 3/4 cytopenia or nephrotoxicity was seen. Two patients (10%) developed anaemia (CTCAE grade 1), while two patients (10%) developed thrombocytopaenia (CTCAE grade 2). One patient received two cycles of half-standard dose (3.7 GBq) of PRRT due to baseline thrombocytopaenia (baseline platelet counts of 87 × 10^9^/L) but received no further doses of PRRT therapy due to concern regarding ongoing and persistent thrombocytopaenia (platelet counts 65 × 10^9^/L to 80 × 10^9^/L). Five patients (25%) experienced fatigue (CTCAE grade 1), four patients (20%) experienced bone pain (CTCAE grade 1–2), two patients (10%) experienced nausea (CTCAE grade 1) and one patient (5%) had hiccoughs post-PRRT therapy. All side-effects were transient. A summary of the side-effects and changes in anti-hypertensives pre and post PRRT is shown in Table 3 and Supplemental Table 3.

**Table 3:** Clinical features (side effects) and changes in antihypertensives pre and post PRRT.

Use of anti-hypertensives, *n* (%)	
Pre PRRT	
Patients on antihypertensives	12 (60%)
Patients not on antihypertensives	8 (40%)
Post PRRT	
Patients on antihypertensives	12 (60%)
Patients not on antihypertensives	8 (40%)
Changes in anti-hypertensives post PRRT, *n* (%)	
Increased	3 (15%)
No changes	17 (85%)
Number of patients with PFNMN levels, *n* (%)	
Pre-PRRT	
Elevated PFNMN	8 (40%)
Normal PFNMN	6 (30%)
PFNMN not available	6 (30%)
Post PRRT	
Elevated PFNMN	6 (30%)
Normal PFNMN	5 (25%)
PFNMN not available	9 (45%)
Minor side effects	
Fatigue	5 (25%)
Bone pain	4 (20%)
Nausea	2 (10%)
Hiccoughs	1 (5%)
Grade 2 anaemia	2 (10%)
Grade 1 thrombocytopaenia	2 (10%)
Major side effects	
Nephrotoxicity	0 (0%)
Grade 3/4 cytopenia	0 (0%)

*n*, number of patients; PRRT, peptide receptor radionuclide treatment; PFNMN, plasma free normetanephrines; SD, standard deviation.

### Survival analysis

**Figure 3:**
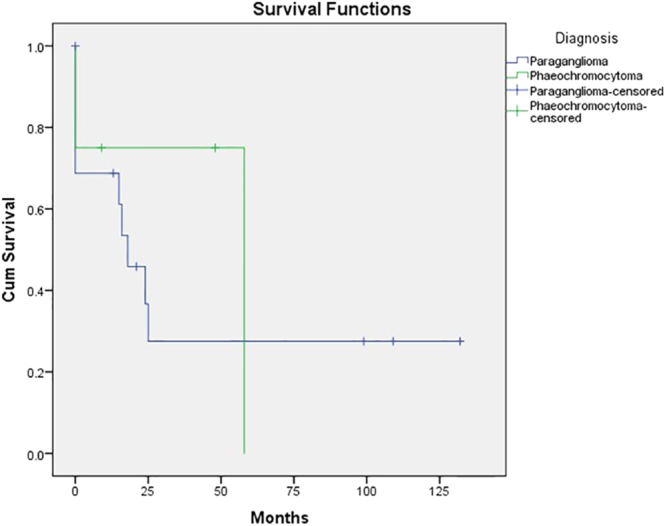
Comparison of progression-free survival (PFS) for phaeochromocytoma (PCC) and paraganglioma (PGL) patients.

Figure 3 shows Kaplan-Meier plots for PFS. Of the total cohort, median PFS from the start of PRRT therapy was 24 months (95% CI: 9.9–38.1). The median PFS for PGL was 18 months (95% CI: 8.4–27.6). OS for the total study cohort and median PFS for PCC were not reached.

**Figure 4:**
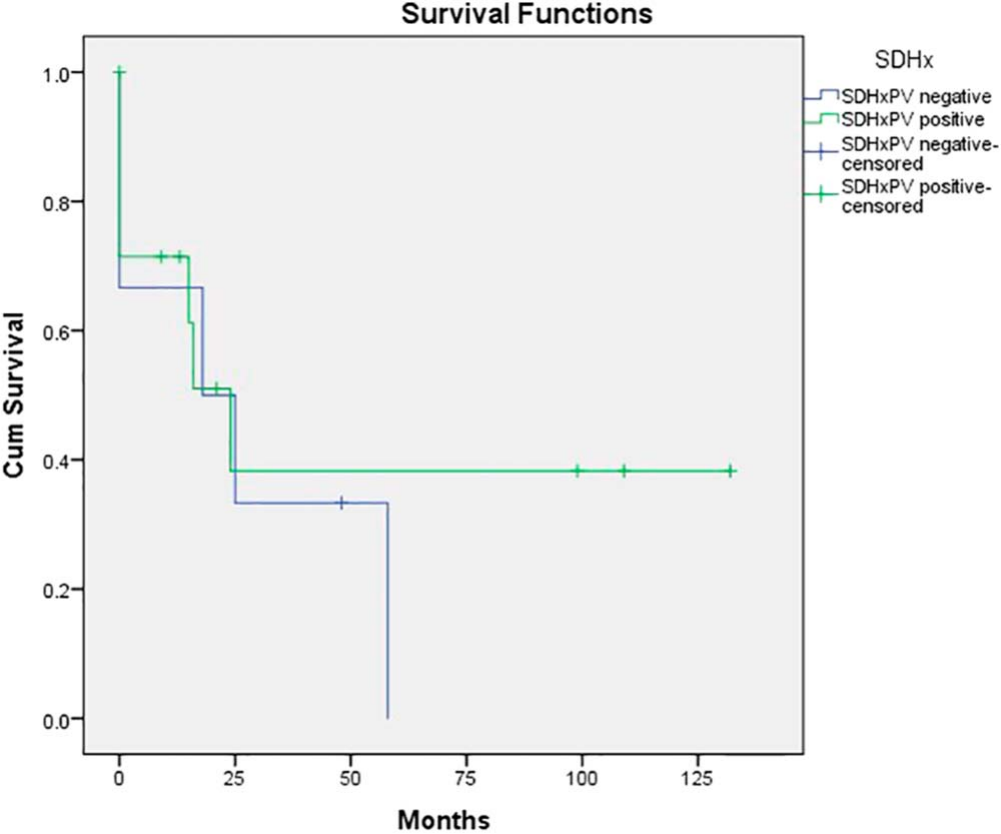
Comparison of progression-free survival (PFS) for *SDHx* positive and negative patient groups.

Of the total cohort of patients, 11 patients (55%) were identified as carriers of *SDHx* PV, whereas six patients (32%) were not. Within the *SDHx* PV-positive subgroup, 60% of patients demonstrated either stable or a partial response to PRRT, whereas in the *SDHx* PV-negative group, 66% of patients experienced stable disease or a partial response to PRRT. The median PFS for those in the *SDHx* PV-positive group was calculated to be 24 months (95% CI: 11.9–36.0), while for those in the *SDHx* PV-negative group, it was calculated to be 18 months (95% CI: 0–48). Figure 4 depicts a comparison of PFS of both groups.

A comparison between *SDHx* PV-positive and negative patients is described in Table 4. As anticipated, *SDHx* PV-negative patients were older than their *SDHx* PV-positive counterparts (*SDHx* PV-positive vs negative: 51 ± 11 years vs 67 ± 13 years, *P*-value = 0.022). There was no significant difference in the rates of hypertension according to *SDHx* PV status (positive vs negative, 60 vs 83%, *P*-value 0.492). Values of PFNMN did not differ pre-treatment but *SDHx* PV-positive versus negative patients had lower PFNMN post-treatment (*SDHx* PV-positive: median 936, IQR 7,340 vs *SDHx* negative: median 28,536, IQR 19,066, *P*-value = 0.032).

**Table 4:** Comparison between *SDHx* PV carriers vs non-*SDHx* group.

Parameter		*P* value (95% CI)
Mean age (years)		
* SDHx* PV positive group	49 ± 12	0.012
* SDHx* PV negative group	67 ± 13	
Tumour type (*n*)		
* SDHx* PV positive group	PGL: 10	0.476
	PCC: 1	
* SDHx* PV negative group	PGL: 3	
	PCC: 3	
Hypertension, *n* (%)		
* SDHx* PV positive group	6 (60%)	0.492
* SDHx* PV negative group	5 (83%)	
Pre-therapy PFNMN, median (min–max, IQR)		
* SDHx* PV positive group	1,124 (408–40,000; 39,309)	0.298
* SDHx* PV negative group	23,169.5 (18,125–31,668; 8,882)	
Post-therapy PFNMN, median (min–max, IQR)		
* SDHx* PV positive group	747 (110–9,404; 5,740)	0.662
* SDHx* PV negative group	28,536 (6,159, 35,500; 19,065.5)	
No of PRRT cycles, median (min–max, IQR)		
* SDHx* PV positive group	4 (2–4; 2)	0.275
* SDHx* PV negative group	4 (2–4; 0)	
Mean cumulative dose of Lu, GBq (mean ± SD)		
* SDHx* PV positive group	22.731 ± 8.923	0.247
* SDHx* PV negative group	27.673 ± 6.032	
Stable disease and partial response, *n* (%) at the end		
* SDHx* PV positive group	7 (64%)	1
* SDHx* PV negative group	4 (67%)	
Progression free survival months (mean ± SD)		
* SDHx* PV positive group	30 ± 38	0.8210
* SDHx* PV negative group	26 ± 25	

Statistical significance was defined as *P* < 0.05. SDHx, succinate dehydrogenase subunits A–B; *n*, number of patients; PGL, paraganglioma; PCC, phaeochromocytoma; PFNMN, plasma free normetanephrines; SD, standard deviation; PRRT, peptide receptor radionuclide therapy; GBq, gigabecquerel; IQR, interquartile range.

### HRQoL analysis

**Figure 5:**
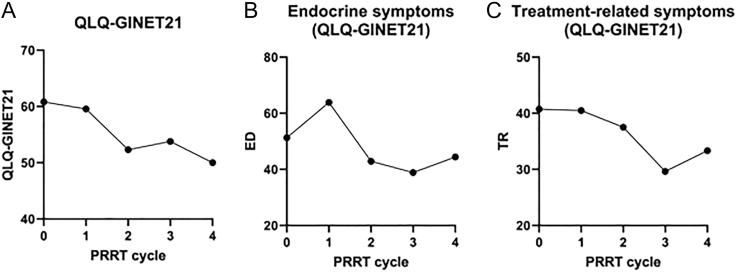
HRQOL scores based on QLQ-GINET21 linear scale before and following each cycle of PRRT (A), endocrine-specific symptoms (ED) (B) and treatment-related symptoms (TR) (C) linear scales throughout PRRT therapy.

The HRQoL analysis, as assessed by the EORTC linear transformation of scores obtained from the QLQ-GINET21 questionnaire, revealed an overall trend of decreasing scores across PRRT cycles (Fig. 5A), indicative of an improvement. The baseline score was 60.8 before the first cycle of PRRT (*n* = 13), with a marginal decrease to 59.6 after the first cycle (*n* = 12) and 52.3 after the second cycle (*n* = 7). For the patients who underwent four cycles of PRRT, the QLQ-GINET21 scores were 53.8 after the third cycle (*n* = 6) and 50.00 after the fourth cycle (*n* = 2).

Endocrine symptoms (ES) initially worsened following the first cycle of PRRT, as indicated by an increase in the linear score from 51.3 at baseline to 63.9 (QLQ-GINET21 items 31–33). However, there was a gradual and sustained improvement in ES severity in subsequent cycles; the reported ES scores decreased to 42.9 and 44.4 following the second and fourth cycles of PRRT, respectively (Fig. 5B). Similarly, treatment-related symptoms (TR) improved across cycles (QLQ-GINET21 items 39–40); the TR score decreased from 40.48 after the first cycle to 37.5 after the second cycle, and 33.3 after the fourth cycle (Fig. 5C).

## Discussion

In our total patient cohort, the number of male and female participants was comparable (male *n* = 11, female *n* = 9), with more than one-third (*n* = 16, 80%) diagnosed with mPPGL. These patients underwent a median of four cycles of PRRT with a median cumulative dose of 29.7 GBq. The overall median follow-up period from the commencement of PRRT therapy was 29 months (5–134 months). Notably, our findings align with the outcomes of various recent retrospective studies (for a summary, see Supplementary Table 4).

Within our cohort of patients with mPPGL, the DCR was 65%, which is lower than that reported in most other studies (where DCR generally exceeded 80%). In addition, the median PFS in our cohort was 24 months, falling within the lower range of other studies reviewed in Supplemental Table 4 except for one prospective phase II clinical trial in which an interim analysis of the safety and efficacy of ^177^Lu-DOTATATE in metastatic or inoperable PPGL patients showed a DCR of >80% in the overall cohort but 72% in patients with *SDHx* PV; the mean PFS was 19.1 months (overall), 22.7 months (sporadic PPGL) and 15.4 months (*SDHx* PV) (21). In a recent review, Pacak and colleagues have summarised meta-analyses and seminal studies carried out in these patients using PRRT (7). The divergence in DCR compared to the existing literature may potentially be attributed to a combination of factors, including one patient who experienced disease progression after PRRT due to receiving only two cycles at a reduced dose (half dose) of ^177^Lu-DOTATATE, which was necessitated by pre-existing thrombocytopaenia. Moreover, in our cohort 13 patients (65%) had two or more previous lines of treatment, indicating higher disease burden at baseline compared to studies done previously, as shown in Supplemental Table 4. Remarkably, 75% of patients with phaeochromocytoma (PCC) showed stable disease following PRRT. Although some of the patients with progressive disease at the start of the PRRT had received other treatments (e.g. chemotherapy) before PRRT therapy, it is unlikely to have had any additive effects on the outcome as the disease progressed after receiving other treatments before PRRT was offered. It is also essential to acknowledge that our study cohort was limited in terms of the patient sample size. Of note, after a detailed analysis of the radiological behaviour of the disease, we observed that our patients commonly exhibited a non-significant *increase* in the size of bone and liver lesions post-PRRT. Whether this is of pathological concern or represents ‘pseudo-progression’ (i.e. the radiological appearance of progression when lesions undergo necrosis/apoptosis) is unclear. On the contrary, the size of peritoneal lesions *regressed* non-significantly following PRRT treatment. Although we did not routinely use molecular imaging for the assessment of treatment response post-PRRT, there is ongoing debate on whether molecular imaging would be better compared to cross-sectional imaging to assess treatment response post-PRRT. Recently updated nuclear medicine guidelines recommend SSTR-PET scan to be done 9–12 months post-PRRT to serve as a new baseline molecular imaging scan for future comparisons (22). In addition, the future use of quantitative measures such as serial or whole-body uptake parameters and changes in volumetric data in molecular/functional imaging will likely serve as important biomarkers for restaging, but further evaluation and prospective studies are warranted (23).

Analysis of the toxicity profile of PRRT in our cohort showed that PRRT therapy was well tolerated by patients. None of the patients developed grade 3 or 4 cytotoxicity. Compared to other studies on mPPGL, this was in the lower half of the range (grade 3 or 4 cytotoxicity: 0–30%) (Supplemental Table 4). None of the patients developed grade 3 or 4 nephrotoxicity, which is comparable to other studies (Supplementary Table 4). It is noted that studies on PRRT therapy for GEP-NEN show that it is associated with 5–10% risk of CTCAE grade 3/4 bone marrow toxicity (24). Only three patients experienced an increase in blood pressure post-PRRT, which is also comparable with the previous studies (25). In six patients, PFNMN levels increased after PRRT, and of these six patients, two patients had progressive disease. A recent study showed a transient increase in plasma catecholamine (noradrenaline, adrenaline, dopamine and normetanephrine) especially 24 and 48 h post-PRRT therapy, but the levels returned to baseline by day 30 (26). None of our patients with secretory mPPGL developed tumour lysis syndrome or a catecholaminergic crisis after PRRT. It is worth noting that all patients with secretory mPPGL received adequate α-adrenoceptor blockade before PRRT therapy, which could have masked any catecholaminergic related changes.

We were unable to highlight a difference in the rate of disease control between *SDHx* positive and negative patients (60 vs 66%), while the median PFS appeared to be somewhat higher in the *SDHx*-negative group (*SDHx* positive versus negative, median PFS 21 vs 45 months), although this was not statistically significant. Our results indicate that PRRT treatment efficacy with regards to disease control is comparable between PV carriers and non-carriers, although our numbers are small. One study evaluated the effect of ^90^Y-DOTATATE therapy in mPPGL related to *SDHx* PVs (25). However, to our knowledge, there is no current comparative study done to compare the effect of ^177^Lu-DOTATATE PRRT therapy in positive and negative groups for genetic PVs.

In addition to the observed disease effects, PRRT in our mPPGL cohort demonstrated an overall improvement in reported symptoms throughout therapy, as evidenced by the increase in QLQ-GINET21 scores. Previous studies have established that PRRT improves HRQoL in patients with GEP-NENs (27, 28, 29). However, the literature on HRQoL in this specific patient population remains limited. Yadav *et al.* reported an improvement in HRQoL scores in paraganglioma patients undergoing PRRT in combination with capecitabine therapy (30). Notably, an initial exacerbation of endocrine symptoms was observed, followed by an improvement from baseline with subsequent treatment cycles. The reason for the initial exacerbation of endocrine symptoms with treatment is not completely understood. Furthermore, the reported treatment-related symptoms underscore the tolerability and safety profile of PRRT in this cohort, a finding consistent with previous studies involving other NET subtypes (31).

### Limitations

The current study has notable limitations that need to be taken into account when interpreting its findings. The primary limitation is the small sample size which, coupled with the retrospective study design, has restricted the statistical power and generalisability of the results. Furthermore, missing values of PFMN at various follow-up time points prohibited us from a pairwise comparison. Hence, the role of PRRT in the catecholamine-secreting capacity of mPPGL could not be fully assessed. From the cohort of patients who progressed through the PRRT treatment, one patient received two cycles of half the standard dose of ^177^Lu-DOTATATE and they had extensive disease burden at the start of the therapy. Finally, given the retrospective nature of this study, we could not reliably confirm administration of specific bone agents to this cohort of patients for those eligible to receive this type of treatment. Furthermore, there were significant limitations in the HRQoL analysis, including a limited sample size and a low questionnaire completion rate, particularly in later PRRT cycles. As a result, meaningful statistical analysis was not feasible and a narrative analysis was performed instead. The QLQ-GINET21 is commonly used to assess HRQoL in NET patients; however, it is important to note that it has been validated in the GEP-NEN cohort, and there is currently no specific validated questionnaire for patients with mPPGL. While the QLQ-C30 remains a reasonable alternative, it was not utilised at the time of the study in accordance with local guidelines. In addition, although not formally validated for this cohort, the EORTC QLQ-H&N might have been a suitable alternative for assessing HRQoL in patients with head and neck paragangliomas.

## Conclusions

PRRT, employing ^177^Lu-DOTATATE PRRT, has been shown to be a safe and effective method for managing metastatic or inoperable PPGL with low toxicity and an encouraging PFS. Considering radiological changes evidenced during disease, PRRT appears to sufficiently control peritoneal metastatic spread but the effect on the progression of disease in the bones remains unclear. It also appears to improve HRQoL in patients with mPPGL. Our experience is concordant with the currently available evidence on the effectiveness of PRRT in mPPGL, suggesting that it should be seriously considered as first-line treatment for patients with slowly to moderately growing m-PPGL with moderate to high tumour burden, considering the fact that it is extremely difficult to conduct and complete prospective clinical trials in this rare tumour that rarely metastasises and requires systemic therapy (7, 32, 33, 34, 35). A phase II prospective trial of ^177^Lu-DOTATATE (*Lutathera*®) PRRT for unlicensed indications is currently recruiting patients which include patients with mPPGL with an estimated completion by the end of 2027 (NCT06121271). In addition, newer targeted therapeutic agents, either alone or in combination with PRRT, are being explored to treat these rare and complex tumours.

## Supplementary materials



## Declaration of interest

The authors declare that there is no conflict of interest that could be perceived as prejudicing the impartiality of the work reported.

## Funding

This work did not receive any specific grant from any funding agency in the public, commercial or not-for-profit sector.

## Author contribution statement

Kalyan Mansukhbhai Shekhda contributed to writing the original draft, reviewing and editing, and statistical analysis. Eleni Armeni was responsible for writing review and editing, statistical analysis and supervision. Yiwang Xu, Shaunak Navalkissoor, Christos Toumpanakis, Dalvinder Mandair, Aimee Hayes, Dominic Yu, Ann-Marie Quigley, Gopinath Gnanasegaran, Ashley B Grossman and Martyn Caplin helped in writing review and editing. Manfredi D’afflitto was responsible for writing review and editing, and statistical analysis. Bernard Khoo contributed to conceptualization, supervision, writing review and editing.

## Data availability

The data that support the findings of this study are available from the corresponding author upon reasonable request.

## Ethical approval statement

As this study was a retrospective audit of practice, ethical approval was not required under the UK Policy Framework for Health and Social Care Practice. Audit registration number: RFH_23/24678.
