# Supplementary material for: 177Lu-DOTATATE peptide receptor radionuclide therapy (PRRT) in metastatic phaeochromocytomas and paragangliomas (mPPGL): a single centre retrospective analysis of experience at an ENETS Centre of Excellence
Source: Endocr Oncol. 2025 Oct 21;5(1):e250019. doi: 10.1530/EO-25-0019 (PMC12550225; doi:10.1530/EO-25-0019)
Supplement: Supplementary file 1 [file supplementary_materials.pdf]

Supplementary Files:

Supplementary Figure 1: EORTC NET-specific questionnaire QLQ-GINET21*

ENGLISH

EORTC QLQ – GI.NET21

Patients sometimes report that they have the following symptoms or problems. Please indicate the extent to which you have experienced these symptoms or problems during the past week. Please answer by circling the number that best applies to you.

During the past week:		Not at all	A little	Quite a bit	Very much
31. Did you have hot flushes?		1	2	3	4
32. Have you noticed or been told by others that you looked flushed/red?		1	2	3	4
33. Did you have night sweats?		1	2	3	4
34. Did you have abdominal discomfort?		1	2	3	4
35. Did you have a bloated feeling in your abdomen?		1	2	3	4
36. Have you had a problem with passing wind/gas/flatulence?		1	2	3	4
37. Have you had acid indigestion or heartburn?		1	2	3	4
38. Have you had difficulties with eating?		1	2	3	4
39. Have you had side-effects from your treatment? *(If you are not on treatment please circle N/A)*	N/A	1	2	3	4
40. Have you had a problem from repeated injections? *(If not having injections please circle N/A)*	N/A	1	2	3	4
41. Were you worried about the tumour recurring in other areas of the body?		1	2	3	4
42. Were you concerned about disruption of home life?		1	2	3	4
43. Have you worried about your health in the future?		1	2	3	4
44. How distressing has your illness or treatment been to those close to you?		1	2	3	4
45. Has weight loss been a problem for you?		1	2	3	4
46. Has weight gain been a problem for you?		1	2	3	4
47. Did you worry about the results of your tests? *(If you have not had tests please circle N/A)*	N/A	1	2	3	4
48. Have you had aches or pains in your muscles or bones?		1	2	3	4
49. Did you have any limitations in your ability to travel?		1	2	3	4
**During the past four weeks:**					
50. Have you had problems receiving adequate information about your disease and treatment?		1	2	3	4
51. Has the disease or treatment affected your sex life (for the worse)? *(If not applicable please circle N/A)*	N/A	1	2	3	4

© QLQ-G.I.NET21 Copyright 2013 EORTC Quality of life Group. All rights reserved.

***permission taken and granted to use this questionaries for non-commercial publication purposes from EORTC Quality of life Group.**

Supplemental Table 1: Baseline characteristics of the study cohort											
**Case no.**	**Sex**	**Age at the start of therapy**	**Primary tumour**	**Previous treatment history**	**Indication for PRRT**	**Site of metastasis**	**Secretory status**	**Number of PRRT cycles**	**Cumulative dose of PRRT (GBq)**	**Follow up (in months)**	**Germline PV**
**1**	**Male**	72	**PCC**	**Surgery, MIBG**	**PD**	**Lung,Liver, Lymphnodes**	**N/D**	4	29.831	**59**	**Negative**
**2**	**Male**	61	**PCC**	**Nil**	**Inoperable disease**	**Adrenal, Bone**	**S**	2	14.890	5	**SDH-B**
**3**	**Female**	69	**PGL**	**Surgery**	**PD**	**Liver, Bone**	**S**	2	15.805	**75**	**SDH-B**
**4**	**Male**	73	**PGL**	**Surgery, CVD, sorafenib, SSTAs**	**PD**	**Bone**	**S**	4	30.583	28	**Negative**
**5**	**Female**	44	**PGL**	**Surgery, radiotherapy**	**PD**	**Bone**	**N/D**	4	29.728	134	**SDH-B**
**6**	**Female**	84	**PGL**	**Nil**	**Inoperable disease**	**Lymph nodes, bone**	**S**	4	29.930	29	**Negative**
**7**	**Male**	60	**PGL**	**Surgery, radiotherapy**	**PD**	**Peritoneum, Bone, Liver**	**NS**	4	22.464	21	**ND**
**8**	**Female**	61	**PGL**	**Surgery, SSTA**	**PD**	**Liver, Peritoneal**	**S**	4	29.687	132	**ND**
**9**	**Female**	35	**PGL**	**Surgery**	**PD**	**Lung, Bone**	**NS**	4	29.775	109	**SDH-B, SDH-D**
**10**	**Female**	63	**PGL**	**Surgery, MIBG therapy**	**PD**	**Liver, Bone**	**S**	2	14.987	13	**SDH-B**
**11**	**Male**	47	**PGL**	**Surgery, 98-Yttrium Octreate therapy**	**PD**	**Lymphnode, Peritoneum, Bone**	**S**	4	29.841	15	**SDH-B**
**12**	**Male**	49	**PGL**	**Surgery, SSTA**	**PD**	**Bone**	**NS**	4	30.670	82	**SDH-C**
**13**	**Male**	44	**PGL**	**Surgery,**	**PD**	**Skeletal**	**N/D**	4	31.026	31	**SDH-B**

				**Radiotherapy**		**lesions**					
**14**	**Female**	61	**PGL**	**Surgery, MIBG therapy, Sunitinib, CapTem**	**PD**	**Lymphnode, Peritoneum**	**N/D**	2	15.516	29	**SDH-B**
**15**	**Male**	49	**PCC**	**Surgery**	**PD**	**Lymphnodes, Peritoneum**	**S**	4	30.994	**72**	**Negative**
**16**	**Female**	72	**PGL**	**Surgery, SSTAs, radiotherapy**	**PD**	**Bone**	**S**	4	28.548	10	**ND**
**17**	**Male**	55	**PGL**	**Surgery**	**PD**	**Adrenal, Peritoneal**	**N/D**	2	15.419	5	**Negative**
**18**	**Female**	77	**PCC**	**Surgery**	**PD**	**Lung, Lymphnode**	**S**	4	29.278	**48**	**Negative**
**19**	**Male**	49	**PGL**	**Surgery, CVD, SSTAs**	**PD**	**Adrenal, Lung**	**NS**	2	7.400	15	**SDH-B**
**20**	**Male**	30	**PGL**	**CVD chemotherapy, SSTAs**	**Inoperable disease**	**Pelvic mass**	**NS**	4	30,404	29	**SDH-B**

Supplemental Table 2: Radiological features and RECIST assessment of the study cohort:

Case number	Indication for PRRT	Site of metastases	SLD change percentage post PRRT Mean±SD (%) (6 ± 20)	Non-target lesion status post PRRT n (%) Progressed n:4(21%) Stable n:14 (74%) Disappeared n:1 (5%)	New lesion? n:7 (37%)	Post PRRT RECIST assessment [Progressive disease n:7 (37%) Stable disease n:11 (58%) Partial response n:1 (5%)]
1	**Progressive disease**	**Lung,Liver, Lymph nodes**	52%	Progressed	Yes	Progressive disease
2	**Inoperable disease**	**Adrenal, Bone**	17%	Stable	No	Stable disease
3	**Progressive disease**	**Liver, Bone**	-2%	Stable	No	Stable disease
4	**Progressive disease**	**Bone**	-17%	Stable	No	Stable disease
5	**Progressive**	**Bone**	-12%	Stable	No	Stable disease
6	**Inoperable disease**	**Lymph nodes, bone**	36%	Stable	No	Stable disease
7	**Inoperable disease**	**Peritoneum, Bone, Liver**	20%	Stable	No	Stable disease
8	**Progressive disease**	**Liver, Peritoneal**	-1%	Stable	No	Stable disease
9	**Progressive disease**	**Lung, Bone**	-31%	Stable	No	Partial response
10	**Progressive disease**	**Liver, Bone**	10%	Stable	No	Stable disease
11	**Progressive disease**	**Lymphnode, Peritoneum, Bone**	6%	Stable	Yes	Progressive disease
12	**Progressive disease**	**Bone, Lymph nodes**	136%	Stable	Yes	Progressive disease
13	**Progressive disease**	**Skeletal lesions**	0%	Stable	No	Stable disease
14	**Progressive disease**	**Lymph node, Peritoneum**	-3%	Disappeared	Yes	Progressive disease
15	**Progressive disease**	**Lymph nodes, Peritoneum**	-7%	Stable	No	Stable disease
16	**Progressive disease**	**Bone**	0%	Progressed	Yes	Progressive disease
17	**Progressive disease**	**Adrenal, Peritoneal**	52%	Progressed	Yes	Progressive disease
18	**Progressive disease**	**Lung, Lymph node**	-9%	Stable	No	Stable disease

19	**Progressive disease**	**Adrenal, Lung**	14%	Progressed	Yes	Progressive disease
20	**Progressive disease**	**Pelvic mass**	-16%	Stable	No	Stable disease

Supplemental Table 3: Clinical features (side effects) and changes in antihypertensives pre and post PRRT:

Case number	AntiHTN (before therapy)	AntiHTN (after therapy)	Changes in antihypertensive therapy	Plasma normetanephrines		Side effects (after therapy)		
				Baseline	Post PRRT	Nephro toxicity	Cyto penia	Others
1	Candesartan 12mg	Candesartan 16mg	Increased	Not available	Not available	Nil	Nil	Fatigue, nausea
2	Propranolol 40mg<br>Candesartan 8mg<br>Phenoxybenzamine 20mg	Propranolol 40mg<br>Candesartan 8mg<br>Phenoxybenzamine 20mg	No change	Not available	Not available	Nil	Nil	Hiccups
3	Doxazocin 2mg	Doxazocin 2mg	No change	Elevated	Elevated	Nil	Nil	Fatigued, tremors, Shingles
4	Amlodipine 10mg	Amlodipine 10mg<br>Doxazocin 16mg	Increased	Elevated	Elevated	Nil	Nil	-
5	Doxazocin 8mg<br>Atenolol 100mg	Doxazocin 8mg<br>Atenolol 100mg	No change	Not available	Normal	Nil	Nil	-
6	Amlodipine 5mg<br>Atenolol 50mg<br>Doxazocin 2mg	Amlodipine 5mg<br>Atenolol 50mg<br>Doxazocin 2mg	No change	Elevated	Elevated	Nil	Nil	-
7	No Antihypertensives	No Antihypertensives	No change	Normal	Normal	Nil	Nil	-
8	No Antihypertensives	No Antihypertensives	No change	Elevated	Not available	Nil	Nil	Pain
9	No Antihypertensives	No Antihypertensives	No change	Normal	Normal	Nil	Nil	-
10	Phenoxybenzamine 30mg<br>Propranolol 20mg	Phenoxybenzamine 30mg	No change	Elevated	Not available	Nil	Nil	Fatigue, pain

		Propranolol 20mg						
11	Doxazocin 1mg	Doxazocin 1mg	No change	Not available	Elevated	Nil	Nil	Fatigue
12	No Antihypertensives	No Antihypertensives	No change	Normal	Not available	Nil	Nil	-
13	No Antihypertensives	No Antihypertensives	No change	Not available	Normal	Nil	Nil	Nil
14	Phenoxybenzamine 40mg<br>Propranolol 40mg<br>Losartan 100mg<br>Lercanidipine 10mg	Phenoxybenzamine 40mg<br>Propranolol 40mg<br>Losartan 100mg<br>Lercanidipine 10mg	No change	Not available	Not available	Nil	Nil	Low platelets
15	Bisoprolol 2.5mg<br>Doxazocin 4mg	Bisoprolol 2.5mg<br>Doxazocin 4mg	No change	Elevated	Elevated	Nil	Nil	Fatigue, nausea
16	Bisoprolol<br>Doxazocin<br>Furosemide	Not available	Not available	Elevated	Not available	Nil	Nil	Neck pain
17	No Antihypertensives	No antihypertensives	No change	Not available	Not available	Nil	Nil	Bony pain, Constipation, sickness
18	Phenoxybenzamine 10mg<br>Doxazocin 3mg	Phemoxybenzamine 40mg<br>Doxazocin 4mg	Increased	Elevated	Elevated	Nil	Nil	Hair loss
19	No antihypertensives	No antihypertensives	No change	Normal	Not available	Nil	Nil	Nil
20	No antihypertensives	No antihypertensives	No change	Normal	Normal	Nil	Nil	Fatigue, Diarrhoea

**Supplementary Table 4: Comparison between various studies of Peptide Receptor Radionuclide Therapy (PRRT) in Phaeochromocytomas and paragangliomas (PPGL)**									
	Year	Number of patients	Type of study	Bone metastases at baseline	*SDHx* PV status	Response rate	Survival (months)	Follow up duration (months)	Grade 3 / 4 Toxicity
Kolasinka-Cwikla et al [1]	2019	Total: 13<br>PCC: 0<br>PGL: 13	PS	70%	*SDHx* positive: 13	CR: 0%<br>PR: 8%<br>SD: 75%<br>PD: 17%<br>DCR: 92%	Median OS: 68<br>Median PFS: 35	48	Nephrotoxicity: 0%<br>Bone marrow toxicity: 15%
Zandee WT et al [2]	2019	Total: 30<br>PCC: 3<br>PGL: 27	RS	N/A	*SDHx* positive: 16<br>*SDHx* negative: 7<br>NA: 7	CR: 0%<br>PR: 23%<br>SD: 67%<br>PD: 10%<br>DCR: 90%	Median OS: NR<br>Median PFS: 30	52.5	Nephrotoxicity: NA<br>Bone marrow toxicity: 20%
Kong G et al [3]	2017	Total: 20<br>PCC: 8<br>PGL: 11<br>Organ of Zuckerkandl: 1	RS	N/A	*SDHx* positive: 8<br>*SDHx* negative: 2<br>NA: 10	CR: 0%<br>PR: 29%<br>SD: 50%<br>PD: 14%<br>DCR: 795	Median OS: NR<br>Median PFS: 39	28	Nephrotoxicity: 0%<br>Bone marrow toxicity: 30%
Severi S et al[4]	2021	Total: 46<br>PCC: NA<br>PGL: NA	Phase II clinical trial	41%	*SDHx* positive: 20<br>*SDHx* negative: 16<br>NA: 10	CR: 0 %<br>PR: 9%<br>SD: 72%<br>PD: 19%<br>DCR: 80%	Median OS: 143.5<br>Median PFS: NR	73	Nephrotoxicity: 0%<br>Bone marrow toxicity: 0%
Jaiswal SK et al[5]	2020	Total: 15<br>PCC: 5<br>PGL: 10	RS	40%	*SDHx* positive: 2<br>*SDHx* negative: 3<br>NA: 10	CR: 0%<br>PR: 7%<br>SD: 73%<br>PD: 20%<br>DCR: 80%	Median OS: NR<br>Median PFS: NR	26	Nephrotoxicity: 0%<br>Bone marrow toxicity: 0%

Vyakaranam A.R. et al [6]	2019	Total: 22<br>PCC: 9<br>PGL: 13	RS	77%	*SDHx* positive: 7<br>*SDHx* negative: 6<br>NA: 9	CR: 0%<br>PR: 9%<br>SD: 91%<br>PD: 0%<br>DCR: 100%	Median OS: 50<br>Median PFS: 22	32	Nephrotoxicity: 0%<br>Bone marrow toxicity: 0%
Nastos et al[7]	2017	Total: 9<br>PCC: 1<br>PGL: 8	RS	78%	*SDHx* positive: 4<br>NA: 5	CR: 0%<br>PR: NA<br>SD: NA<br>PD: NA<br>DCR: NA	Median OS: 60<br>Median PFS: 39	39	Nephrotoxicity: 0%<br>Bone marrow toxicity: 22%
Mitjavila M et al[8]	2022	Total: 31<br>PCC: NA<br>PGL: NA	RS	65%	NA: 31	CR: 0%<br>PR: 19%<br>SD: 65%<br>PD: 15%<br>DCR: 85%	Median OS: NR<br>Median PFS: 31	NA	Nephrotoxicity: NA<br>Bone marrow toxicity: NA
Fischer A et al[9]	2023	Total: 22<br>PCC: NA<br>PGL: NA	RS	NA	*SDHx* positive: 14<br>*SDHx* negative: 6<br>NA: 2	CR: 0%<br>PR: N/A<br>SD: N/A<br>PD: N/A<br>DCR: 67%	Median OS: NA<br>Median PFS: 18	NA	Nephrotoxicity: NA<br>Bone marrow toxicity: NA
Pinato et al[10]	2016	Total: 5<br>PCC: 0<br>PGL: 5	RS	100%	*SDHx* positive: 5<br>*SHDx* negative: 0	CR: 0%<br>PR: 20%<br>SD: 60%<br>PD: 20%<br>DCR: 80%	Median OS: NR<br>Median PFS: 17	NA	Nephrotoxicity: NA<br>Bone marrow toxicity: NA
Hamiditabar et al[11]	2017	Total: 5<br>PCC: 1<br>PGL: 4	PS	NA	NA: 5	CR: 0%<br>PR: 0%<br>SD: 80%<br>PD: 20%<br>DCR: 80%	Median OS: NA<br>Median PFS: NA	NA	Nephrotoxicity: NA<br>Bone marrow toxicity: NA

Demirci et al[12]	2018	Total: 8<br>PCC: NA<br>PGL: NA	RS	NA	NA: 8	CR: 0%<br>PR: 50%<br>SD: 25%<br>PD: 25%<br>DCR: 75%	Median OS: 51.8<br>Median PFS: 31.4	NA	Nephrotoxicity: NA<br>Bone marrow toxicity: NA
Yadav et al*[13]	2019	Total: 25<br>PCC: 0<br>PGL: 25	RS	52%	NA: 25	CR: 0%<br>PR: 28%<br>SD: 56%<br>PD: 16%<br>DCR: 84%	Median OS: NR<br>Median PFS: 32	30	Nephrotoxicity: 0%<br>Bone marrow toxicity: 0%
Roll et al[14]	2020	Total: 7<br>PCC: 0<br>PGL: 7	RS	14%	NA: 7	CR: 0%<br>PR: 0%<br>SD: 100%<br>PD: 0%<br>DCR: 100%	Median OS: NA<br>Median PFS: NA	39	Nephrotoxicity: 0%<br>Bone marrow toxicity: 0%
Parghane et al [15]	2021	Total: 9<br>PCC: 0<br>PGL: 9	RS	NA	NA: 7	CR: 0%<br>PR: 11%<br>SD: 56%<br>PD: 33%<br>DCR: 67%	Median OS: NR<br>Median PFS: NR	40	Nephrotoxicity: 0%<br>Bone marrow toxicity: 0%
Prado-Wohlwend et al[16]	2022	Total: 9<br>PCC: 3<br>PGL: 6	RS	89%	*SDHx* positive: 4<br>*SDHx* negative: 5	CR: 0%<br>PR: 22%<br>SD: 67%<br>PD: 11%<br>DCR: 89%	Median OS: NA<br>Median PFS: 29	25.6	Nephrotoxicity: 0%<br>Bone marrow toxicity: 11%
Tang et al**[17]	2023	Total: 15<br>PCC: 3<br>PGL: 12	RS	80%	*SDHx* positive: 4<br>*SDHx* negative: 4<br>NA: 7	CR: 0%<br>PR: 44.4%<br>SD: 33.3%<br>PD: 22.2%<br>DCR: 77.8%	Median OS: NR<br>Median PFS: 25.9§	54	Nephrotoxicity: 7%<br>Bone marrow toxicity: 67%

Our study	2025	Total: 20<br>PCC: 4<br>PGL: 16	RS	55%	*SDHx* positive: 11<br>*SDHx* negative: 6<br>NA: 3	CR: 0%<br>PR: 5%<br>SD: 60%<br>PD: 35%<br>DCR: 65%	Median OS: NR<br>Median PFS: 24	29	Nephrotoxicity: 0%<br>Bone marrow toxicity: 0%

PCC, phaeochromocytomas; PGL: paragangliomas; PS, prospective study; RS, retrospective study; NA, not available; *SDHx*, Succinate dehydrogenase subunits A-B; CR, complete response; PR, partial response; SD, stable disease; PD, progressive disease; DCR, disease control rate; OS, overall survival; PFS, progression free survival.

*Treated with ^{177}Lu-DOTATATE and capecitabine combined therapy

** Response to treatment was studied in 11 patients out of 15 included in the study.

References:

[1] Kolasinska-Ćwikła A, Pęczkowska M, Ćwikła J, Michałowska I, Pałucki J, Bodei L, et al. A Clinical Efficacy of PRRT in Patients with Advanced, Nonresectable, Paraganglioma-Pheochromocytoma, Related to SDHx Gene Mutation. J Clin Med 2019;8:952. https://doi.org/10.3390/jcm8070952.

[2] Zandee WT, Feelders RA, Smit Duijzentkunst DA, Hofland J, Metselaar RM, Oldenburg RA, et al. Treatment of inoperable or metastatic paragangliomas and pheochromocytomas with peptide receptor radionuclide therapy using 177Lu-DOTATATE. Eur J Endocrinol 2019;181:45–53. https://doi.org/10.1530/EJE-18-0901.

[3] Kong G, Grozinsky-Glasberg S, Hofman MS, Callahan J, Meirovitz A, Maimon O, et al. Efficacy of Peptide Receptor Radionuclide Therapy for Functional Metastatic Paraganglioma and Pheochromocytoma. J Clin Endocrinol Metab 2017;102:3278–87. https://doi.org/10.1210/jc.2017-00816.

[4] Severi S, Bongiovanni A, Ferrara M, Nicolini S, Di Mauro F, Sansovini M, et al. Peptide receptor radionuclide therapy in patients with metastatic progressive pheochromocytoma and paraganglioma: long-term toxicity, efficacy and prognostic biomarker data of phase II clinical trials. ESMO Open 2021;6:100171. https://doi.org/10.1016/j.esmoop.2021.100171.

[5] Jaiswal SK, Sarathi V, Memon SS, Garg R, Malhotra G, Verma P, et al. 177Lu-DOTATATE therapy in metastatic/inoperable pheochromocytoma-paraganglioma. Endocr Connect 2020;9:864–73. https://doi.org/10.1530/EC-20-0292.

[6] Vyakaranam AR, Crona J, Norlén O, Granberg D, Garske-Román U, Sandström M, et al. Favorable Outcome in Patients with Pheochromocytoma and Paraganglioma Treated with 177Lu-DOTATATE. Cancers (Basel) 2019;11:909. https://doi.org/10.3390/cancers11070909.

[7] Nastos K, Cheung VTF, Toumpanakis C, Navalkissoor S, Quigley A-M, Caplin M, et al. Peptide Receptor Radionuclide Treatment and (131)I-MIBG in the management of patients with metastatic/progressive phaeochromocytomas and paragangliomas. J Surg Oncol 2017;115:425–34. https://doi.org/10.1002/jso.24553.

[8] Mitjavila M, Jimenez-Fonseca P, Belló P, Pubul V, Percovich JC, Garcia-Burillo A, et al. Efficacy of [177Lu]Lu-DOTATATE in metastatic neuroendocrine neoplasms of different locations: data from the SEPTRALU study. Eur J Nucl Med Mol Imaging 2023;50:2486–500. https://doi.org/10.1007/s00259-023-06166-8.

[9] Fischer A, Kloos S, Remde H, Dischinger U, Pamporaki C, Timmers HJLM, et al. Responses to systemic therapy in metastatic pheochromocytoma/paraganglioma: a retrospective multicenter cohort study. Eur J Endocrinol 2023;189:546–65. https://doi.org/10.1093/ejendo/lvad146.

[10] Pinato DJ, Black JRM, Ramaswami R, Tan TM, Adjogatse D, Sharma R. Peptide receptor radionuclide therapy for metastatic paragangliomas. Medical Oncology 2016;33:47. https://doi.org/10.1007/s12032-016-0737-9.

[11] Hamiditabar M, Ali M, Roys J, Wolin EM, O'Dorisio TM, Ranganathan D, et al. Peptide Receptor Radionuclide Therapy With 177Lu-Octreotate in Patients With Somatostatin Receptor Expressing Neuroendocrine Tumors. Clin Nucl Med 2017;42:436–43. https://doi.org/10.1097/RLU.0000000000001629.

[12] Demirci E, Kabasakal L, Toklu T, Ocak M, Şahin OE, Alan-Selcuk N, et al. 177Lu-DOTATATE therapy in patients with neuroendocrine tumours including high-grade (WHO G3) neuroendocrine tumours. Nucl Med Commun 2018;39:789–96. https://doi.org/10.1097/MNM.0000000000000874.

[13] Yadav MP, Ballal S, Bal C. Concomitant 177Lu-DOTATATE and capecitabine therapy in malignant paragangliomas. EJNMMI Res 2019;9:13. https://doi.org/10.1186/s13550-019-0484-y.

[14] Roll W, Müther M, Sporns PB, Zinnhardt B, Suero Molina E, Seifert R, et al. Somatostatin Receptor–Targeted Radioligand Therapy in Head and Neck Paraganglioma. World Neurosurg 2020;143:e391–9. https://doi.org/10.1016/j.wneu.2020.07.165.

[15] Parghane R V., Talole S, Basu S. 131I-MIBG negative progressive symptomatic metastatic paraganglioma: response and outcome with 177Lu-DOTATATE peptide receptor radionuclide therapy. Ann Nucl Med 2021;35:92–101. https://doi.org/10.1007/s12149-020-01541-z.

[16] Prado-Wohlwend S, Del Olmo-García MI, Bello-Arques P, Merino-Torres JF. [177Lu]Lu-DOTA-TATE and [131I]MIBG Phenotypic Imaging-Based Therapy in Metastatic/Inoperable Pheochromocytomas and Paragangliomas: Comparative Results in a Single Center. Front Endocrinol (Lausanne) 2022;13:778322. https://doi.org/10.3389/fendo.2022.778322.

[17] Tang CYL, Chua WM, Huang HL, Lam WW, Loh LM, Tai D, et al. Safety and efficacy of peptide receptor radionuclide therapy in patients with advanced pheochromocytoma and paraganglioma: A single-institution experience and review of the literature. J Neuroendocrinol 2023;35. https://doi.org/10.1111/jne.13349.
